# Integrated Bioinformatic Analysis Reveals the Oncogenic, Survival, and Prognostic Characteristics of TPX2 in Hepatocellular Carcinoma

**DOI:** 10.1007/s10528-024-10840-3

**Published:** 2024-06-04

**Authors:** Weibin Zhang, Jia Dong, Yunfei Wu, Xiangnan Liang, Lida Suo, Liming Wang

**Affiliations:** 1 Department of Hepatobiliary Surgery, The Second Hospital of Dalian Medical University, Dalian, China; 2 Department of Radiology, Jinzhou Maternity and Infant Hospital, Jinzhou, China; 3 Department of General Surgery, Jinzhou Central Hospital, Jinzhou, China

**Keywords:** TPX2, Prognosis, Hepatocellular carcinoma, TP53 mutation, Immune infiltration, Co-expression

## Abstract

Targeting protein for Xenopus kinesin-like protein 2 (TPX2), a well-known mitotic protein, has been linked to carcinogenesis in several cancers. This study investigated the role of TPX2 in hepatocellular carcinoma (HCC) from various aspects using bioinformatic analyses. TPX2 expression and its prognostic value in pan-cancers were analyzed using SangerBox. TPX2 expression and its association with prognosis, immune infiltration, tumor mutations, and signaling pathways in HCC were analyzed using UALCAN, BoxKaplan-Meier Plotter, GEPIA, Human Protein Atlas, TIMER 2.0, and SangerBox. Genes co-expressed with TPX2 in HCC were analyzed using the HCCDB database, followed by functional enrichment using SangerBox. Clinical predictive models were established based on TPX2 and its co-expressed genes using the ACLBI database. TPX2 expression significantly increased in pan-cancers and was associated with survival in nearly half of the cancer types. High TPX2 expression has been linked to poor survival outcomes in patients with HCC. TPX2 expression was positively correlated with abundant infiltration of immune cells (including B cells, CD4 + /CD8 + T cells, macrophages, neutrophils, and dendritic cells), TP53 mutation, and carcinogenesis-related pathways, such as the PI3K/AKT/mTOR pathway, cellular response to hypoxia, and tumor proliferation signature. Nineteen genes were found to be co-expressed with TPX2 in HCC, and these genes showed close positive correlations and were mainly implicated in cell cycle-related functions. A prognostic model established using TPX2 and its expressed genes could stratify HCC patients into high- and low-risk groups, with a significantly shorter survival time in high-risk groups. The prognostic model performed well in predicting 1-, 3-, and 5-year survival of patients with HCC, with areas under the curve of 0.801, 0.725, and 0.711, respectively. TPX2 functions as an oncogene in HCC, and its high expression is detrimental to the survival of patients with HCC. Thus, TPX2 may be a prognostic biomarker and potential therapeutic target for HCC.

## Introduction

Liver cancer is a common malignancy and the third leading cause of cancer-related deaths worldwide, with an estimated 905,677 new cases (4.7%) and 830,180 deaths (8.3%) reported in the 2020 global cancer statistics (Sung et al. 2021). Approximately 72.5% of liver cancers worldwide are reported in Asian countries, and China accounts for the majority of these cases in Asia (Zhang et al. 2022), where the major etiology is chronic hepatitis B virus (HBV) infection (Yang et al. 2023). Hepatocellular carcinoma (HCC) is the main histological type of primary liver cancer, accounting for approximately 75%-85% of all cases (Ganesan and Kulik 2023). Currently, liver resection is the preferred treatment for HCC. Nevertheless, most HCC patients miss the opportunity for surgery because of their middle and late stages of diagnosis, and even after surgery, these patients often have diminished clinical outcomes due to a high rate of recurrence and distant metastasis (Liu and Song 2021; Zhou and Song 2021). Thus, it is important to identify new prognostic markers for monitoring and predicting tumor recurrence and prognosis, which may contribute to the clinical trends of patients with HCC.

Targeting protein for Xenopus kinesin-like protein 2 (TPX2) is a protein-coding gene first identified in African clawed toad eggs that promotes chromatin microtubule nucleation, regulates spindle formation, and is closely related to the proliferation of microtubules in the nucleus (King and Petry 2020; Bird and Hyman 2008). Increasing evidence has demonstrated the close involvement of TPX2 in multiple malignant tumors (such as esophageal, prostate, lung, breast, and gastric cancers) (Zou et al. 2018a; Sui et al. 2019; Yang et al. 2015; Tomii et al. 2017; Zhou et al. 2020; Neumayer et al. 2014). It is frequently highly expressed in cancers and may play an oncogenic role in modulating various cancer-associated cellular events such as epithelial-mesenchymal transition (Zhang et al. 2021), cell migration and invasion (Yang et al. 2015), and tumor metastasis and resistance (Hu et al. 2023).

Several studies have reported the involvement of TPX2 in HCC (Wang et al. 2022a; Wang et al. 2023a; Huang et al. 2019). For example, TPX2 expression is elevated in HCC and its silencing inhibits the malignant behavior of tumor cells (Huang et al. 2019). In another study, TPX2 expression was found to be reduced in HCC-infiltrating CD8 + T cells, and downregulated TPX2 restricted the anticancer effect of CD8 + T cells in HCC, whereas such antitumor activity was enhanced by TPX2 overexpression (Wang et al. 2022a). These findings suggest that the exact role of TPX2 in HCC remains unclear. Therefore, this integrated bioinformatic analysis was performed to uncover the expression, prognostic value, and potential biological functions of TPX2 in the development and progression of HCC.

## Methods

The bioinformatics analyses in this study were conducted using online tools or online platforms, and all the analyses were conducted at January 2023.

### SangerBox Analysis

SangerBox 3.0 (http://vip.sangerbox.com/) is a powerful online platform integrating various databases for interactive bioinformatics (Shen, et al. 2022). Based on data from TCGA and Genotype Tissue Expression (GTEx) databases, differences in TPX2 expression in tumor vs. normal tissues were explored using the SangerBox portal. The COX_OS (overall survival) analysis of TPX2 levels in pan-cancer and the relationship between TPX2 expression and the gene mutation landscape were analyzed using Sangerbox. Additionally, the functional enrichment of TPX2 and its co-expressed genes was analyzed using Sanger box.

### UALCAN Database Analysis

UALCAN (http://ualcan.path.uab.edu.) (Chandrashekar et al. 2017) is an interactive web portal for analyzing cancer omics data. Expression of TPX2 in HCC tumors and normal tissues and its expression among HCC subgroups divided by TP53 mutation status, tumor stages, and grades were evaluated based on the UALCAN database.

### Kaplan–Meier Plotter

The Kaplan–Meier plotter (http://kmplot.com/analysis/) (Győrffy 2023) is a web database for evaluating the associations between gene expression and survival in diverse tumor types, and the used data was derived from the Gene Expression Omnibus (GEO), European Genome-Phenome Archive (EGA), and TCGA databases. Using a Kaplan–Meier plot, correlations between TPX2 expression and overall survival (OS), progression-free survival (PFS), disease-specific survival (DSS), and recurrence-free survival (RFS) in HCC were evaluated. Hazard ratios (HRs) with 95% confidence intervals (CI) and P values were determined using Cox proportional hazards regression, followed by the log-rank test.

### Gene Correlation Analysis in GEPIA

The GEPIA database (http://gepia.cancer-pku.cn/) (Tang et al. 2017) is an interactive web tool capable of assessing the RNA-seq data of 9,736 tumors and 8,587 normal samples derived from TCGA and GTEx databases. Correlations between TPX2 and TP53 expression were analyzed using GEPIA and correlation coefficients were determined using the Spearman method.

### HPA Database Analysis

The Human Protein Atlas (HPA; https://www.proteinatlas.org/) (Thul and Lindskog 2018) is an open-access database that provides immunohistochemistry (IHC)-based protein expression profiles of cell lines, cancer, and normal tissues. The protein levels of TPX2 in HCC and normal liver tissues were explored based on IHC data from the HPA database.

### TIMER Analysis

TIMER2.0 (http://timer.cistrome.org/) is a web server that provides comprehensive analysis of tumor-infiltrating immune cells (Li, et al. 2020). Spearman’s correlation between TPX2 and immune cell infiltration levels and tumor purity was investigated using the Gene Module provided in TIMER2.0. The overall survival between groups stratified by TPX2 expression level in HCC was assessed utilizing the “Outcome Module” of IMER2.0.

### HCCDB Database Analysis

HCCDB is an online database developed based on 15 publicly available HCC expression datasets covering approximately 4000 clinical samples and is used to explore gene expression in HCC (Lian et al. 2018). Genes co-expressed with TPX2 were analyzed using the HCCDB database.

### ACLBI Database Analysis

The web-based tool Assistant for Clinical Bioinformatics (ACLBI, https://www.aclbi.com/) was used to evaluate the prognostic power of TPX2 in patients with HCC based on TCGA level 3 RNA-seq and clinical data. HCC tumor samples were separated into high and low TPX2 expression groups according to their median values, and the differences in overall survival probability between the two TPX2 expression groups were analyzed by survival analysis; hazard ratios with 95% confidence intervals and log-rank P values were determined. Furthermore, the independent prognostic power of TPX2 and its co-expressed genes was investigated using univariate and multivariate Cox regression analyses, and a nomogram was developed according to the identified independent prognostic genes. LASSO Cox regression analysis was conducted for TPX2 and its co-expressed genes to identify a prognostic signature, and a prognostic model was developed. Patients with HCC were stratified into low- and high-risk groups, and OS was compared between the groups. The predictive accuracy of TPX2 and/or the prognostic model for 1-, 3-, and 5-year survival of patients with HCC were assessed using timeROC (v 0.4) analysis. Additionally, pathway scores were analyzed using ssGSEA, and associations between gene expression and pathway scores were explored using Spearman’s correlation analysis.

## Results

### Expression of TPX2 in *Pan*-Cancers

**Fig. 1:**
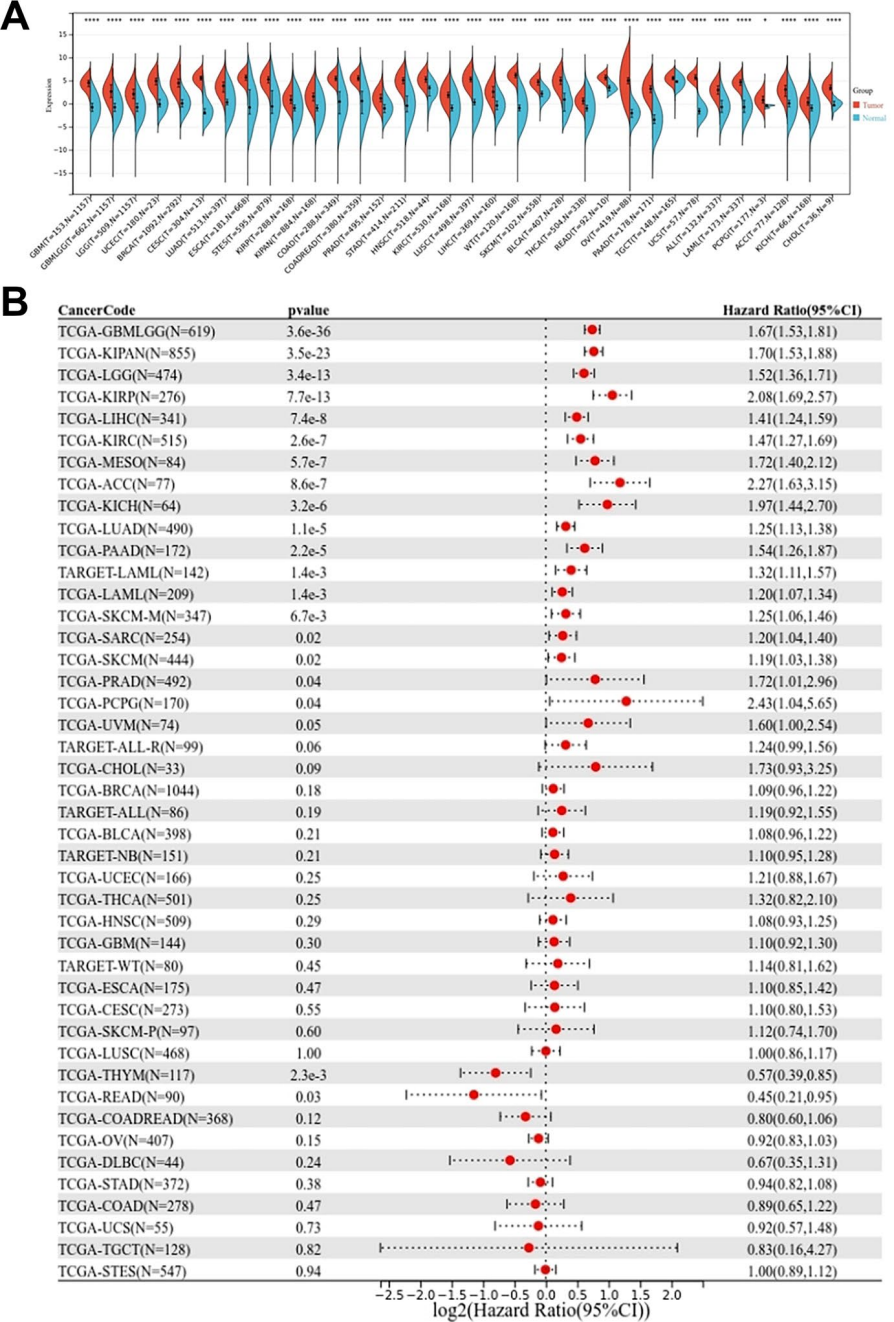
Expression and prognosis value of TPX2 in pan-cancers. **A**, Expression of TPX2 across different cancer types analyzed using SangerBox, *P<0.05, ****P<0.0001. **B**, Forest plot showing the prognostic value of TPX2 for different cancer types using univariate cox survival analysis, with P<0.05 representing statistical significance.

Expression and prognostic value of TPX2 in pan-cancers were analyzed firstly, and the abbreviations and full names of these tumor types are shown in Table 1. The expression pattern of TPX2 across different cancer types was explored using the SangerBox portal. Significantly elevated expression of TPX2 was observed in all cancer types (Fig. 1A), indicating that TPX2 has a potential oncogenic role. The prognostic power of TPX2 in pan-cancers was further revealed, and TPX2 was found to be markedly associated with OS in nearly half of the cancer types (Fig. 1B). Among these cancer types, high TPX2 expression may be a risk factor for poor prognosis in most cancers, such as adrenocortical cancer (ACC, HR = 2.27, p = 8.6e-7), kidney renal papillary cell carcinoma (HR = 2.08, p = 7.7e-13), and HCC (HR = 1.41, p = 7.4e-8). In contrast, high TPX2 levels may be a protective factor for the prognosis of patients with thymoma (THYM, HR = 0.57, p = 2.3e-3) and rectal adenocarcinoma (READ, HR = 0.45, p = 0.03).

**Table 1:** Abbreviations and full names of the cancer types in pan-cancers analysis in this study

Cancer code	Full name
TCGA-GBM/LGG	Glioblastoma and lower grade glioma
TCGA-KIPAN	Pan-kidney cohort
TCGA-LGG	Lower Grade Glioma
TCGA-KIRP	Kidney Papillary Cell Carcinoma
TCGA-LIHC	Liver Cancer
TCGA-KIRC	Kidney Clear Cell Carcinoma
TCGA-MESO	Mesothelioma
TCGA-ACC	Adrenocortical Cancer
TCGA-KICH	Kidney Chromophobe
TCGA-LUAD	Lung Adenocarcinoma
TCGA-PAAD	Pancreatic Cancer
TARGET-LAML	Acute Myeloid Leukemia
TCGA-SKCM-M	Skin Cutaneous Melanoma
TCGA-SARC	Sarcoma
TCGA-SKCM	Melanoma
TCGA-PRAD	Prostate Cancer
TCGA-PCPG	Pheochromocytoma & Paraganglioma
TCGA-UVM	Ocular melanomas
TARGET-ALL-R	Acute Lymphoblastic Leukemia
TCGA-CHOL	Bile Duct Cancer
TCGA-BRCA	Breast Cancer
TARGRT-ALL	Acute Lymphoblastic Leukemia
TCGA-BLCA	Bladder Cancer
TARGET-NB	Neuroblastoma
TCGA-UCEC	Endometrioid Cancer
TCGA-THCA	Thyroid Cancer
TCGA-HNSC	Head and Neck Cancer
TCGA-GBM	Glioblastoma
TARGET-WT	High-Risk Wilms Tumor
TCGA-ESCA	Esophageal Cancer
TCGA-CESC	Cervical Cancer
TCGA-LUSC	Lung Squamous Cell Carcinoma
TCGA-THYM	Thymoma
TCGA-READ	Rectal Cancer
TCGA-COAD/READ	Colon and Rectal Cancer
TCGA-OV	Ovarian Cancer
TCGA-DLBC	Large B-cell Lymphoma
TCGA-STAD	Stomach Cancer
TCGA-COAD	Colon Cancer
TCGA-UCS	Uterine Carcinosarcoma
TCGA-TGCT	Testicular Cancer
TCGA-STES	Stomach and Esophageal carcinoma

TCGA, The cancer genome atlas; TARGET, Therapeutically applicable research to generate effective treatments

### High Expression of TPX2 in HCC

**Fig. 2:**
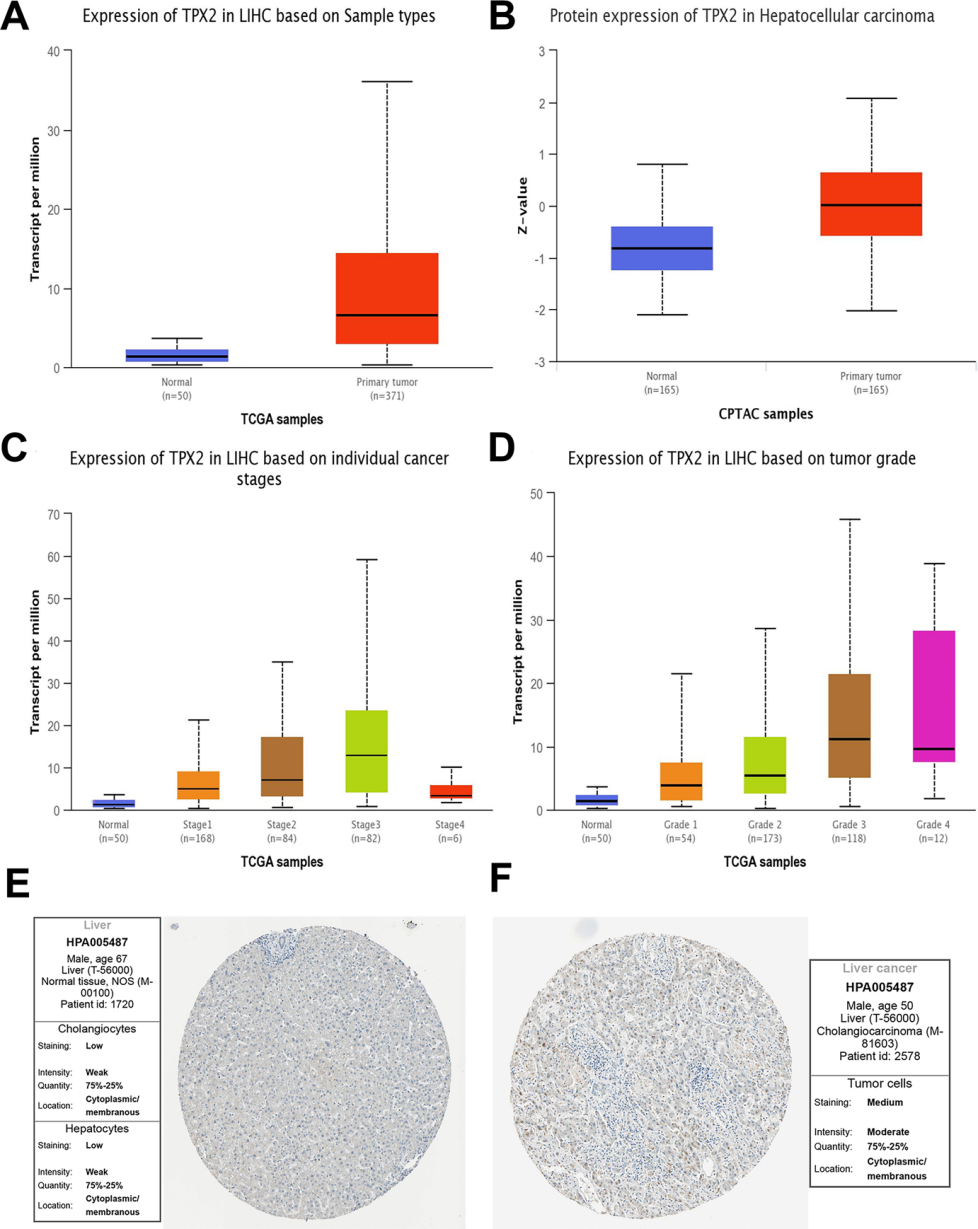
Expression of TPX2 in HCC patients. **A**–**D**, TPX2 expression in the HCC samples was analyzed using the UALCAN database. Expression of TPX2 in HCC tumor and normal control samples at mRNA (**A**) and protein (**B**) levels; **C**–**D**, expression of TPX2 in subgroups divided by tumor stage (**C**) and tumor grade (**D**); **E**–**F**, immunohistochemical staining of TPX2 in normal tissue (**E**) and HCC tumor tissue (**F**) samples analyzed using the human protein atlas database. LIHC, liver hepatocellular carcinoma; TCGA, The Cancer Genome Atlas; CPTAC, Clinical Proteomic Tumor Analysis Consortium.

To investigate the expression pattern of TPX2 in HCC, UALCAN analysis was conducted, and TPX2 was highly expressed in HCC tumor tissues compared to normal controls at both the mRNA and protein levels (Fig. 2A–B). Further analysis revealed the potential association of TPX2 expression with tumor stage and grade. In subgroups divided by tumor stage, expression of TPX2 tended to increase with tumor stage, with the highest TPX2 expression in stage 3 tumors and an obvious decrease in TPX2 expression in stage 4 tumors (Fig. 2C). Such decrease might be explained by the small sample size in stage 4 tumors (*n* = 6, while *n* = 82 in stage 3) and the heterogeneity of clinical samples. Sample size is relatively small and lacks representativeness. Similarly, TPX2 expression tended to increase with tumor grade, with grade 3 tumors showing the highest TPX2 expression (Fig. 2D). Additionally, the IHC data of TPX2 was analyzed based on the HPA database. As shown in Fig. 2E–F, the staining intensity for normal tissue was weak, indicating a low expression of TPX2 in normal tissue. While for tumor tissue, the staining intensity was moderate, indicating an increased expression of TPX2 in tumor tissue. The IHC data confirmed the enhanced level of TPX2 in HCC tumor tissues compared to that in the normal controls. These findings suggested that TPX2 is highly expressed in HCC cells.

### High TPX2 Expression Related to Poor Survival Outcomes in HCC

**Fig. 3:**
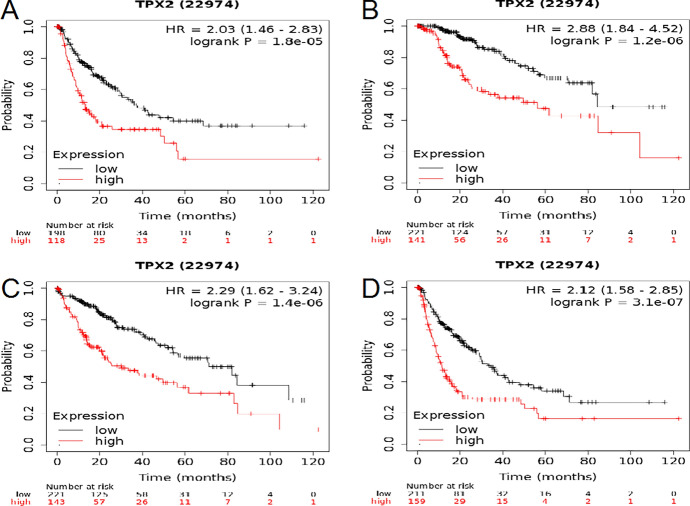
Survival analysis. Kaplan-Meier Plot analysis showing the prognostic association of TPX2 expression with RFS (**A**), DSS (**B**), OS (**C**), and PFS (**D**) in patients with HCC. The log rank test was used for comparisons between high and low expression groups, with P<0.05 representing statistical significance. Hazard ratio and corresponding 95% confidence interval were calculated using univariate Cox regression.

**Fig. 4:**
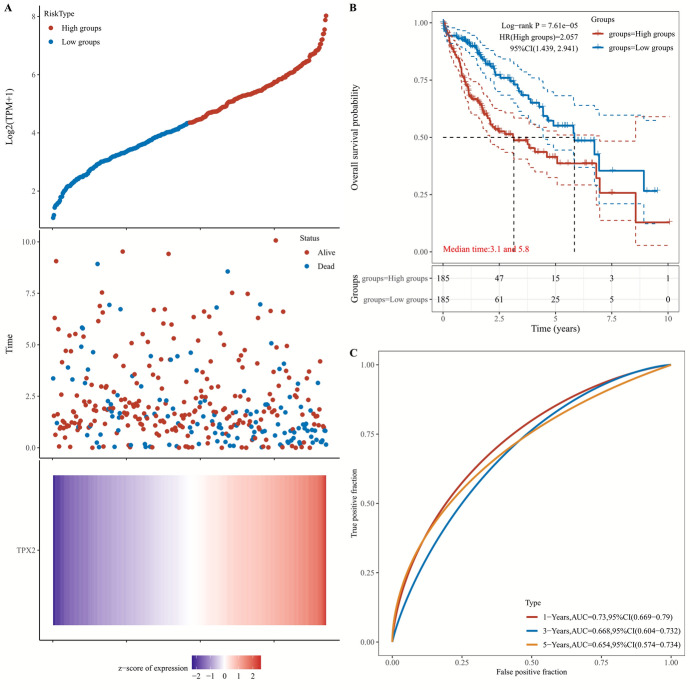
Prognostic value of TPX2 in HCC analyzed by ACLBI analysis. **A**, Sample distribution based on TPX2 expression. HCC samples were divided into high and low TPX2 expression groups based on the median expression value (upper panel), scatterplot showing the sample distribution based on survival status (middle panel), and heat map showing the sample distribution ranked by TPX2 expression (bottom panel); **B**, survival curve showing the prognostic value of TPX2 in HCC. The log rank test was used for comparisons between high and low expression groups, with P<0.05 representing statistical significance; **C**, ROC curves showing the power of TPX2 for predicting the 1-, 3-, and 5-year survival of patients with HCC.

Considering the abnormal expression of TPX2 in patients with HCC, we further explored its prognostic association with HCC. Patients with HCC with high TPX2 expression showed shorter RFS (HR = 2.03, P = 1.8e-05), DSS (HR = 2.88, P = 1.2e-06), OS (HR = 2.29, P = 1.4e-06), and PFS (HR = 2.12, P = 3.1e-07) than those with low TPX2 expression (Fig. 3), suggesting the potential of TPX2 as an indicator of prognosis in HCC. This was confirmed by the ACLBI analysis (Fig. 4). The median survival time of patients with HCC and high TPX2 expression was 3.1 years, which was significantly shorter than the median survival time of 5.8 years in patients with low TPX2 expression (*P* = 7.61e-05). ROC curves indicated that TPX2 had moderate predictive power for 1-, 3-, and 5-year survival of patients with HCC, with areas under the curve of 0.73, 0.668, and 0.654, respectively.

### TPX2 Expression Positively Correlated with Immune Cell Infiltration

**Fig. 5:**
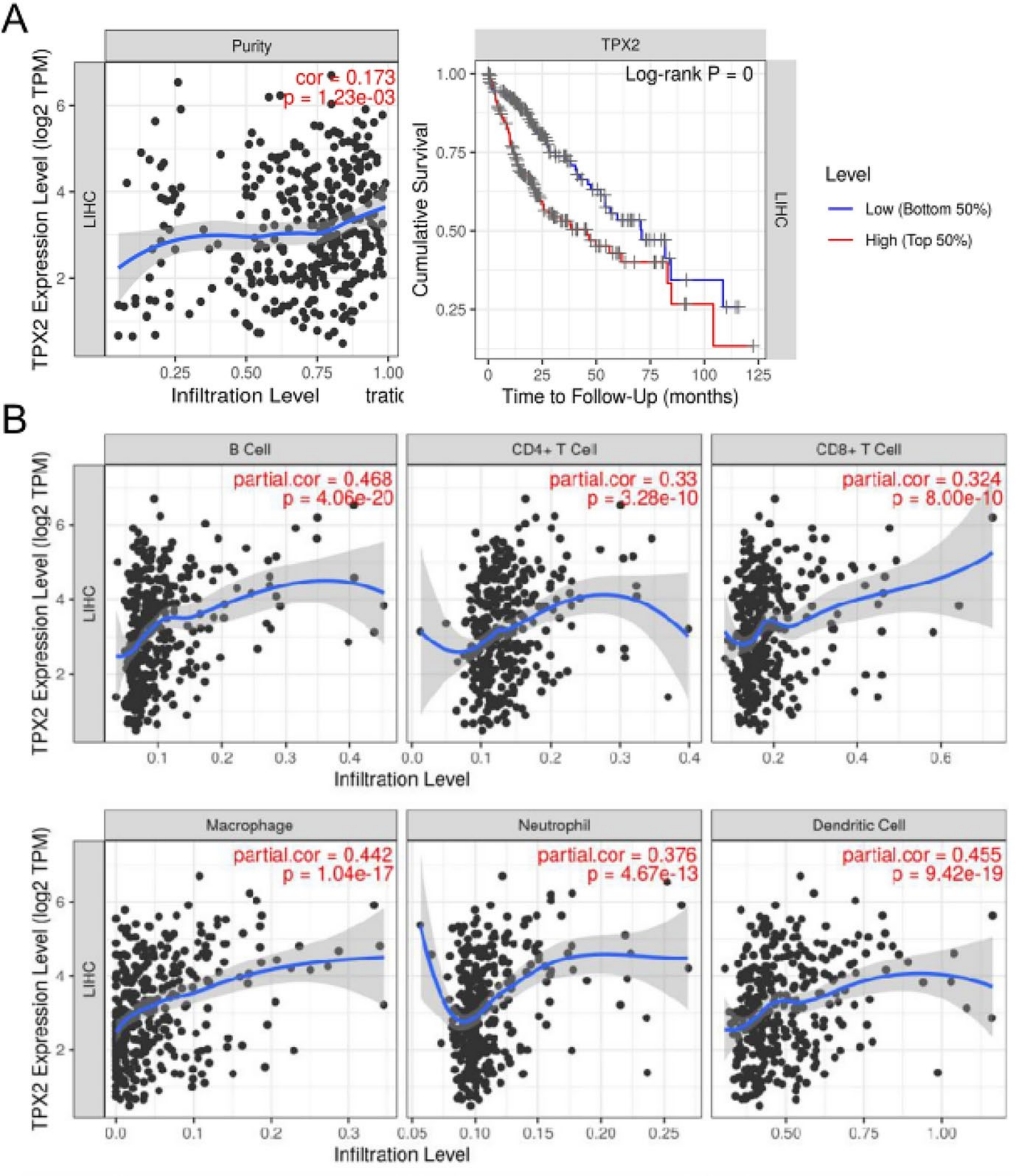
Associations of TPX2 with immune infiltration. **A**, Scatter plot showing the correlation between TPX2 expression and tumor purity (left) and survival curve showing the association of TPX2 expression with cumulative survival (right, log rank P<0.05 representing statistical significance); **B**, Scatter plots showing the correlation between TPX2 expression and the infiltration abundance of six immune cells. The correlation coefficient in scatter plots was calculated using Spearman correlation analysis. LIHC, liver hepatocellular carcinoma

The association between TPX2 expression and the infiltration abundance of the six main immune cell types in the tumor microenvironment of HCC was explored using TIMER 2.0. We found that TPX2 correlated with tumor purity in HCC, and its high TPX2 expression was linked to worse cumulative survival in HCC in the TIMER 2.0 analysis (Fig. 5A). Particularly, the expression of TPX2 showed outstanding positive correlations with the infiltration abundance of all six immune cells, including B cells (*r* = 0.468, *p* = 4.06e-20), macrophages (*r* = 0.442, *p* = 1.04e-17), CD4 + T cells (*r *= 0.33, *p* = 3.28e-10), neutrophils (*r* = 0.376, *p* = 4.67e-13), CD8 + T cells (*r* = 0.324, *p* = 8.00e-10), and dendritic cells (*r* = 0.455, *p* = 9.42e-19) (Fig. 5B). These findings suggest that TPX2 expression is involved in immune infiltration into the HCC tumor microenvironment.

### Expression of TPX2 Positively Correlated with TP53 Mutation

**Fig. 6:**
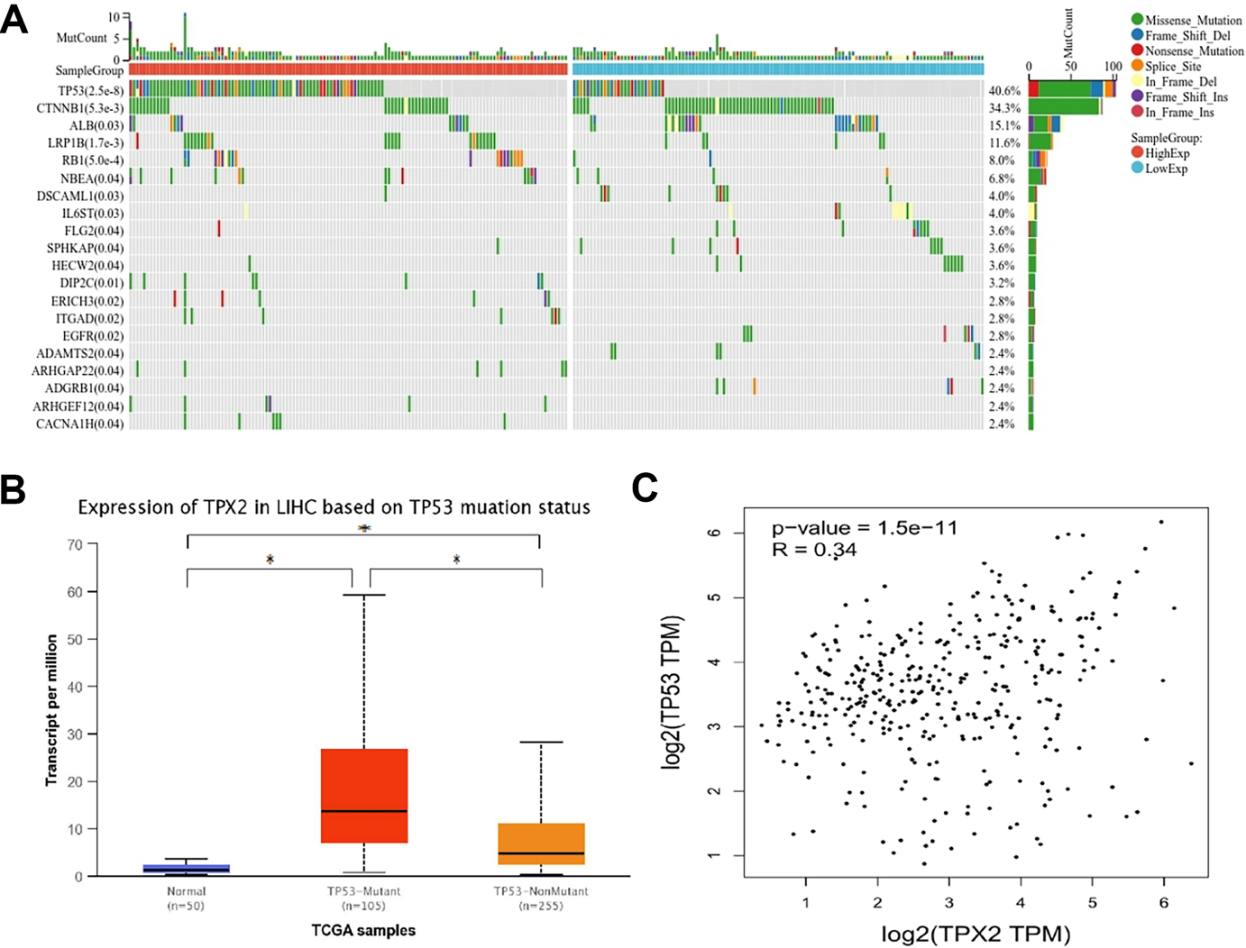
Associations of TPX2 with tumor mutation. **A**, Mutation landscape of HCC samples in the high and low TPX2 expression groups analyzed using SangerBox; **B**, expression of TPX2 in HCC subgroups divided by TP53 mutation status analyzed using the UALCAN database, *P<0.05; **C**, Scatter plot showing the positive correlations between TPX2 expression and TP53 expression analyzed using GEPIA, in which the correlation coefficient in scatter plots was calculated using Spearman correlation analysis.

Gene mutation landscapes of HCC samples in the high and low TPX2 expression groups were visualized using SangerBox (Fig. 6A). Missense mutations accounted for majority of the mutations. TP53 (40.6%) was the most frequently mutated gene in the HCC samples, followed by CTNNB1 (34.3%), ALB (15.1%), and LRPB (11.6%). In particularly, in HCC samples with high TPX2 expression, TP53 mutations occurred in more than half of the samples, which was more frequent than that in HCC samples with low TPX2 expression. The well-known tumor suppressor gene TP53 is frequently mutated in human cancers (Giacomelli et al. 2018); this mutation is one of the most common genetic changes in HCC and is associated with the progression and prognosis of HCC (Li et al. 2022; Yang, et al. 2021). Therefore, we further investigated the expression of TPX2 in HCC subgroups divided by TP53 mutation status and found significantly higher expression of TPX2 in TP53-mutated tumor samples than that in TP53-non-mutated samples (Fig. 6B). GEPIA revealed that TPX2 expression was positively correlated (*r* = 0.34, *P* = 1.5e-11) with TP53 expression in HCC (Fig. 6C).

### TXP2 Expression-Related Signaling Pathways in HCC

**Fig. 7:**
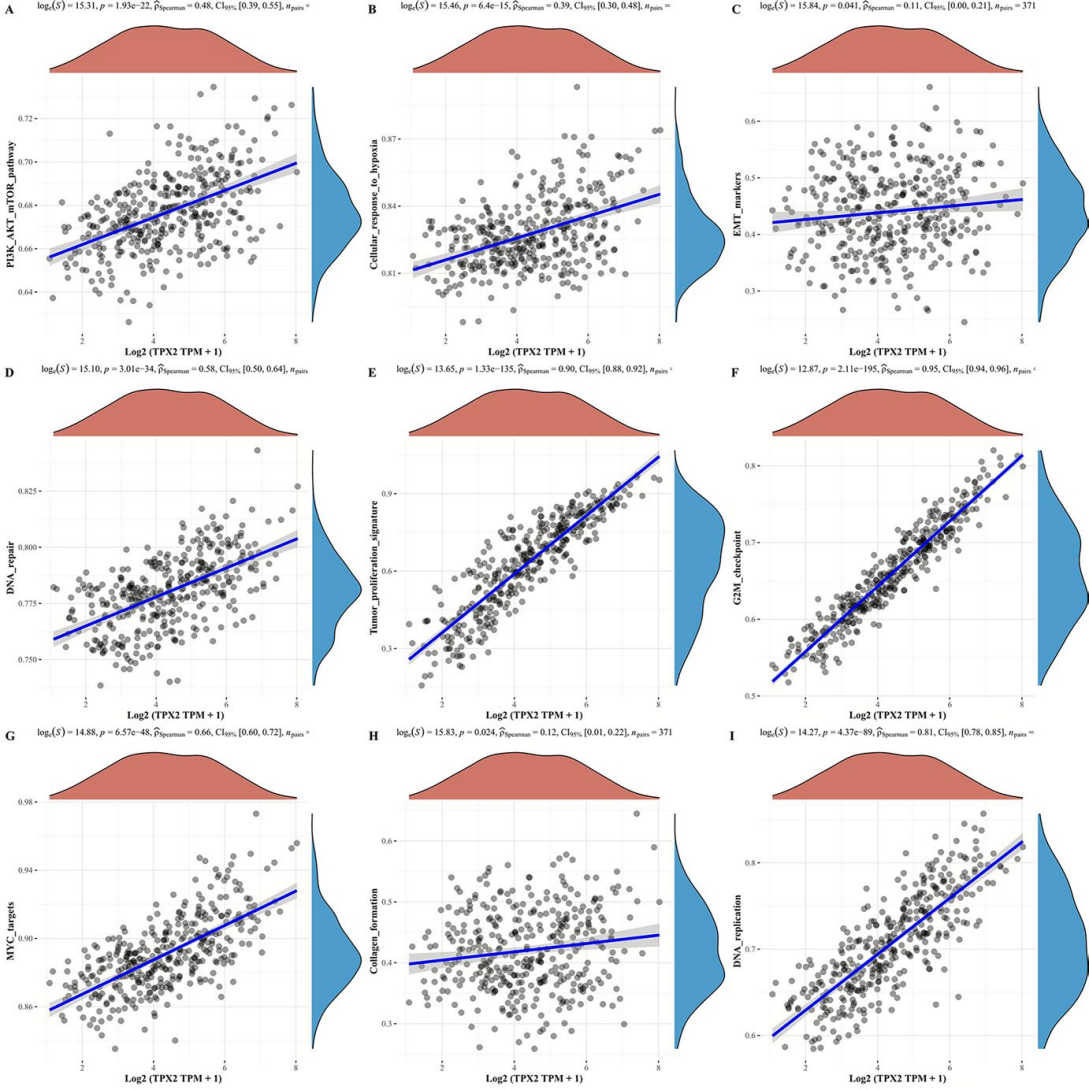
TXP2 expression-related signaling pathways. Scatter plots showing Spearman’s correlations between TXP2 expression and pathway scores analyzed using the ACLBI database, including PI3K_AKT_mTOR pathway (**A**), cellular response to hypoxia (**B**), EMT markers (**C**), DNA repair (**D**), tumor proliferation signature (**E**), G2M checkpoint (**F**), MYC targets (**G**), collagen formation (**H**), and DNA replication (**I**). The x-axis represents the distribution of TPX2 gene expression, and the y-axis represents the distribution of the pathway scores. The Spearman’s correlation coefficient, P value and corresponding 95% confidence interval were shown in the upper of each panel.

We further investigated the signaling pathways that may be influenced by TPX2 expression in HCC. Based on the Spearman correlation provided in ACLBI, we found that TPX2 was markedly positively correlated with multiple carcinogenesis-related pathways or hallmark gene sets (Fig. 7), including the PI3K/AKT/mTOR pathway (*r* = 0.48, *p* = 1.93e-22), cellular response to hypoxia (*r* = 0.39, *p *= 6.4e-15), tumor proliferation signature (*r* = 0.90, *p* = 1.33e-135), G2M checkpoints (*r* = 0.95, *p* = 2.11e-195), EMT markers (*r* = 0.11, *p* = 0.041), collagen formation (*r* = 0.12, *p* = 0.024), MYC targets (*r* = 0.66, *p* = 6.57e-48), DNA damage repair (*r* = 0.58, *p* = 3.01e-34), and DNA replication (*r* = 0.81, *p* = 4.37e-89). In particularly, expression of TPX2 showed strong positive correlations with tumor proliferation signature, G2M checkpoints, DNA damage repair, and DNA replication (all r > 0.5), indicating that tumor proliferation was promoted with increased TPX2 expression.

### Co-Expressed Genes with TPX2 in HCC

**Fig. 8:**
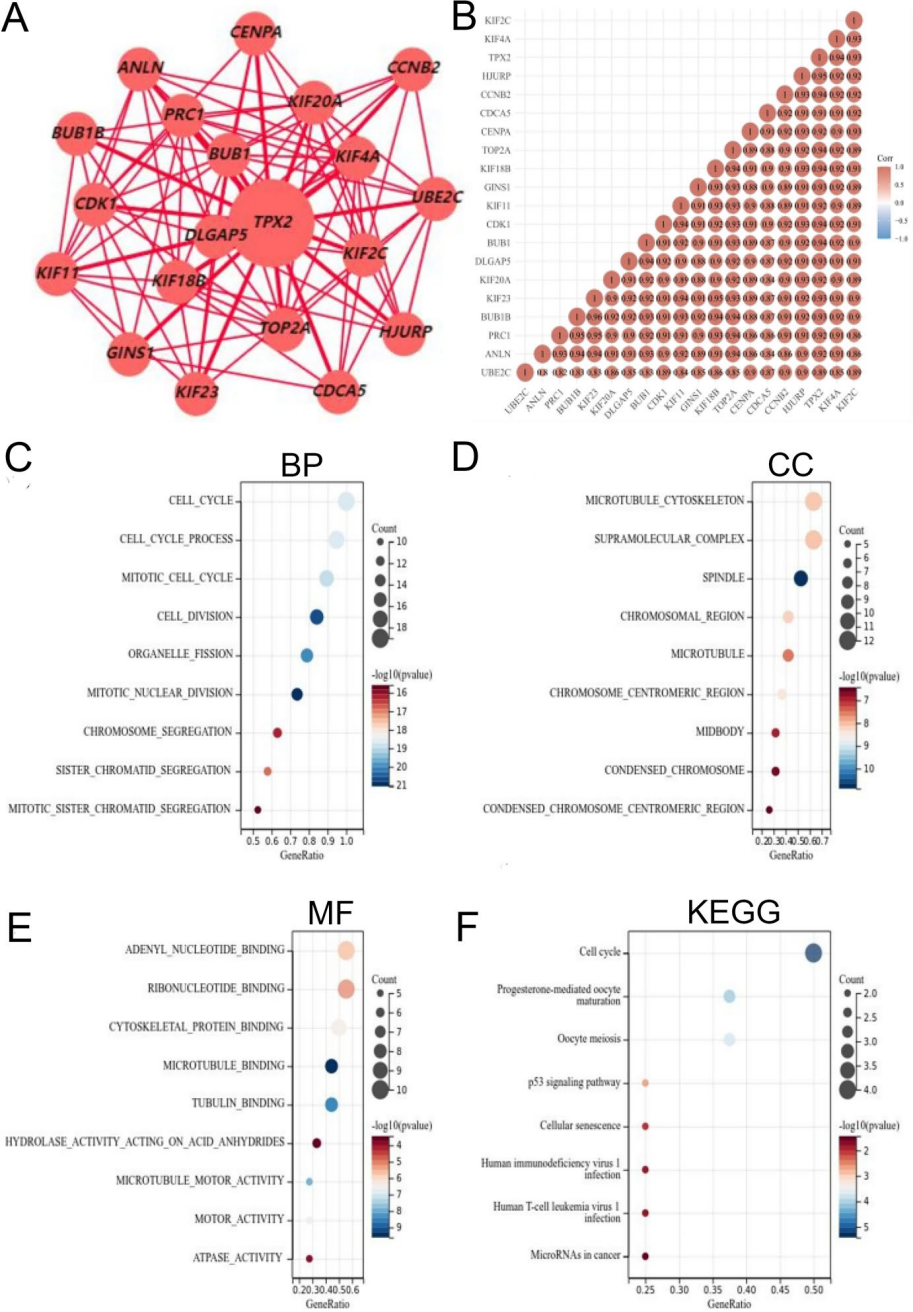
Co-expressed genes with TPX2. **A**, Co-expression network for TPX2 analyzed using the HCCDB database; **B**, Correlation map showing the expression correlations among these co-expressed genes and TPX2. The numbers in each circle are the Spearman’s correlation coefficient. Red and blue colors represent positive and negative correlations, respectively; **C**–**F**, functional enrichment for TPX2 and its co-expressed genes, including gene ontology biological process (**C**), cellular component (**D**), molecular function (**E**), and KEGG pathways (**F**). In the bubble diagram of enrichment analysis, node size represents the count of genes; color from red to blue represents the P value from significant to unsignificant.

To comprehensively explore the possible biological functions of TPX2 in HCC, we analyzed the genes co-expressed with TPX2 using the HCCDB database. Nineteen genes were found to be co-expressed with TPX2 (Fig. 8A), and there were close positive correlations among these genes (Fig. 8B), indicating that these genes might act as functional modules in HCC. Enrichment analyses for TPX2 and its co-expressed genes showed they were mainly implicated in cell cycle-related gene ontology functions, such as mitotic nuclear division, chromosome segregation, cell division biological processes, microtubule formation, midbody, condensed chromosome cellular component and microtubule binding, and microtubule motor activity molecular function (Fig. 8C–E). In addition, these genes were markedly involved in KEGG pathways such as the cell cycle, P53 signaling pathway, and cellular senescence (Fig. 8F).

### Clinical Predictive Models Established by TPX2 and its Co-Expressed Genes

**Fig. 9:**
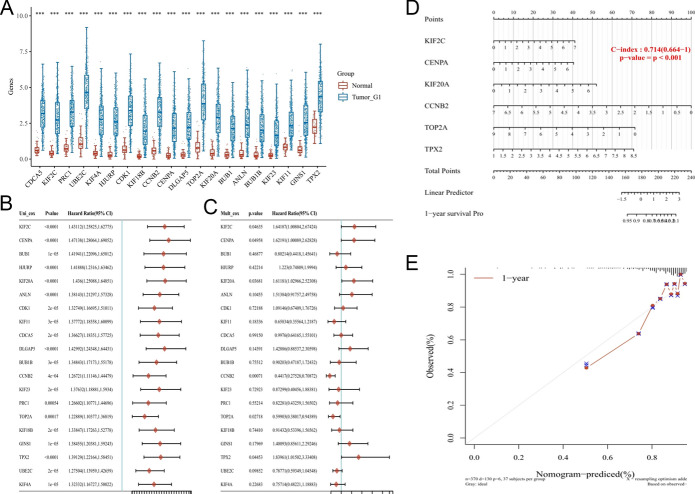
Independent prognostic value of TPX2 and its co-expressed genes. **A**, Boxplots showing the expression of TPX2 and its co-expressed genes in HCC tumors and normal tissues, ***P<0.001; univariate (**B**) and multivariate (**C**) Cox regression for screening the genes that are independently associated with survival of HCC patients, with P<0.05 represent statistical significance. Hazard ratio over 1 represent protective factors, whereas hazard ratio less than 1 represent risk factors; **D**, predictive nomogram for predicting 1-year survival of HCC patients established based on independent prognostic genes; E, calibration curve for assessing the predictive nomogram.

The prognostic value of TPX2 and its co-expressed genes in HCC was analyzed using ACLBI, and we found that all these genes were highly expressed in HCC tumor tissues compared to normal tissues (Fig. 9A), which could also be inferred from the positive correlations among these genes in the co-expression network. Univariate Cox analysis indicated that all these genes were significantly detrimental to the OS of patients with HCC (all p < 0.05; Fig. 9B). Further multivariate Cox analysis identified six genes that were independently associated with the survival of patients with HCC, including TPX2 (HR = 1.84), KIF2C (HR = 1.64), CENPA (HR = 1.62), KIF20A (HR = 1.61), CCNB2 (HR = 0.44), and TOP2A (HR = 0.60) (all p < 0.05, Fig. 9C). Based on these six independent prognostic genes, a predictive nomogram was established that showed good performance in predicting the 1-year survival of patients with HCC (C-index of 0.714, Fig. 9D). Calibration curves indicated high consistency between the estimated and actual survival rates (Fig. 9E).

**Fig. 10:**
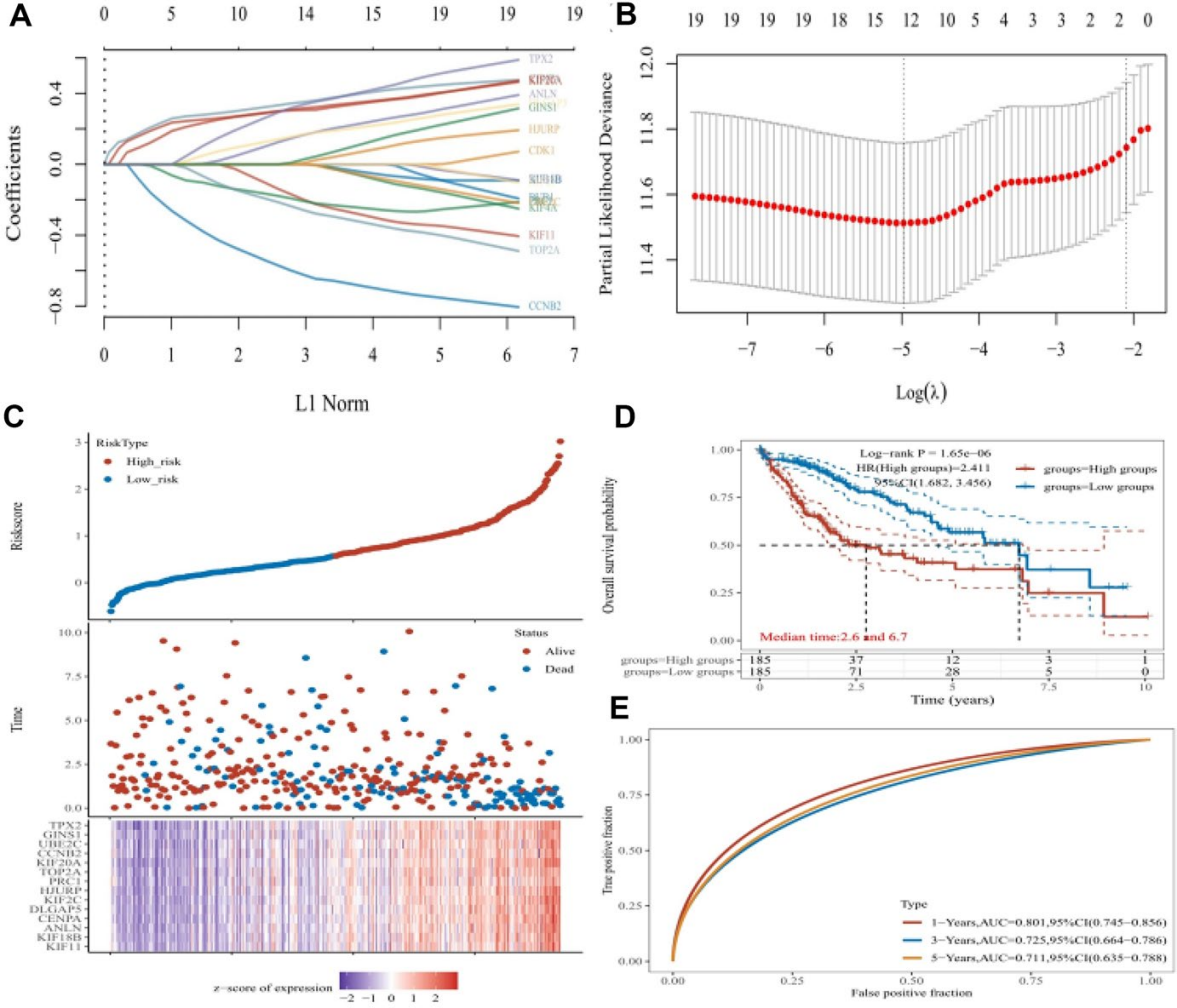
Risk prognostic model. **A**, Distribution of the LASSO coefficient (with 10-fold cross-validation) analyzed using glmnet package; **B**, partial likelihood deviance of LASSO coefficient distribution; **C**, distribution of samples marked by risk score (upper) and survival state (middle), and heat map of gene expression with risk score (bottom); **D**, survival curve showing survival between low- and high-risk groups, and P value was calculated by log-rank test, and hazard ratio and corresponding 95% confidence interval were calculated using univariate Cox regression; E, ROC curves showing the power of the prognostic model for predicting 1-, 3-, and 5-year survival of patients with HCC, which was conducted using the timeROC(v 0.4) package.

A prognostic signature was identified from TPX2 and its co-expressed genes using LASSO analysis, which consisted of 14 genes: TPX2, GINS1, UBE2C, CCNB2, KIF20A, TOP2A, PRC1, HJURP, KIF2C, DLGAP5, CENPA, ANLN, KIF18B, and KEF11 (Fig. 10A–B). A risk prognostic model was established based on these 14 genes, and the risk score for each patient was calculated and used to stratify patients into high- and low-risk groups (Fig. 10C). The expression of these genes increased with an increase in risk score (Fig. 10C). The survival curve indicated that patients in the high-risk group had a short survival time in comparison with those in the low-risk group (median survival time 2.6 vs. 6.7 years, P < 1.65e-06, Fig. 10D). ROC curves (Fig. 10E) indicated that this risk prognostic model performed well in predicting the 1-, 3-, and 5-year survival of patients with HCC, with areas under the curve of 0.801, 0.725, and 0.711, respectively.

## Discussion

HCC is a malignant tumor of the digestive system prompted by both pathogenic and genetic factors (Toh et al. 2023; Vogel et al. 2022). Although great progress has been made on the molecular mechanism and drug therapy in recent years (Awad, et al. 2023; Abdel-Latif et al. 2022; Benassi et al. 2021; Nelson, et al. 2022), the overall prognosis of patients with liver cancer is still unsatisfactory. According to the most recent cancer statistics data, HCC is the third leading cause of cancer death worldwide (Sung et al. 2021), and the second leading cause of cancer death in China (Feng et al. 2023). The causative oncogenes play vital roles in the occurrence of HCC. However, the lack of in-depth research on HCC-related oncogenes and the corresponding targeted therapeutic agents makes it difficult to accurately evaluate patient prognosis (Chen et al. 2019). Hence, it is important to identify potential biomarkers to determine the survival and prognosis of HCC.

TPX2, located on chromosome 20q11.21, mediates microtubule assembly in the mitophagus through its carboxy-terminal domain, and is regulated by Ran-GFP. TPX2 is released early during mitosis to promote the initiation of spindle assembly in concert with AURKA kinase (Wei et al. 2015), a crucial factor in human carcinogenesis (Saeki et al. 2009). TPX2 is closely related to the cell cycle; its expression is observed during the transition from the G1 to S phase, and it disappears during cytoplasmic division (Wadsworth and TPX2. Curr Biol 2015). Reportedly, TPX2 is abnormally expressed in malignancies, and due to the abnormal amplification of TPX2 centrosomes, it often leads to DNA heteroploidy and polyploidy formation, causing massive proliferation and deterioration of tumor cells and affecting the cell cycle and apoptosis (Koike et al. 2022; Zou et al. 2018b). Consistent with these previous studies, the results of correlation analysis in this study also suggested that expression of TPX2 showed strong positive correlations with pathways, including tumor proliferation signature, G2M checkpoints, DNA damage repair, and DNA replication (all r > 0.5), indicating that tumor proliferation was promoted with increased TPX2 expression in HCC. Therefore, abnormal expression of TPX2 is of great value in predicting the development, recurrence, and metastasis of malignant tumors, including HCC.

In the current study, we observed significantly elevated TPX2 expression in all cancer types and its levels were linked to survival outcomes in at least 20 cancer types. Consistent with our findings, previous studies have shown enhanced TPX2 expression in most tumor tissues, which is detrimental to patient survival. For example, in a study of 43 cases of neuroblastoma, TPX2 was found to positively regulate the proliferation, cell cycle, and survival of neuroblastoma cells, which have an oncogenic role in the development of neuroblastoma, indicating the value of TPX2 as a prognostic indicator in neuroblastoma (Koike et al. 2022). Kahl et al. (Kahl et al. 2022) experimentally explored TPX2 expression in 3952 breast cancer cases and observed markedly increased expression of TPX2 in tumor tissues, and its high expression was detrimental to the OS of patients, suggesting that TPX2 could be used as a therapeutic target. Wang et al. (Wang et al. 2022b) investigated changes in the mRNA and protein levels of TPX2 in endometrial cancer tissues and found that enhanced TPX2 expression was related to poor prognosis and was a susceptibility gene for endometrial cancer. Similarly, based on the analysis of 250 cases of esophageal tumor and paracarcinoma tissue, Sui et al. (Sui et al. 2019) showed that the TPX2 level was markedly enhanced in tumor tissues and was unfavorable for the 5-year OS of patients, which was proposed as an important indicator to assess prognosis in esophageal cancer. Additionally, Zhu et al. (Zhu et al. 2020) found that TPX2 expression was elevated in HCC tissues and that its expression was associated with pathological features and clinical outcomes. Therefore, the findings obtained by numerous researchers in different fields agree that high TPX2 expression in tumor tissues increases the prognostic risk for patients and is detrimental to their survival, similarly to the findings of the present study.

The role of TPX2 in chromosome separation during mitosis has been reported to be important for maintaining genomic stability. Evidence suggests that targeting TPX2 in tumor cells could enhance genomic instability, and significant associations between TPX2 and the prognosis of tumors with genomic instability have been proposed (Gijn et al. 2019; Hsu et al. 2017). TP53 mutations are closely associated with increased chromosomal instability, including the deep deletion of tumor suppressor genes and elevated oncogene amplification (Donehower et al. 2019). Based on immunohistochemical staining for TPX2 in 253 primary breast cancer tissues, Matson et al. proposed that higher TPX2 nuclear expression showed outstanding correlations with higher average ploidy, supernumerary centrosomes, and an elevated incidence of TP53 mutations (Matson et al. 2021). The present study indicated that TP53 mutations occurred in more than half of the HCC samples with high TPX2 expression, which was more frequent than in HCC samples with low TPX2 expression. In addition, TPX2 was significantly more highly expressed in the TP53 mutated group than in the TP53 non-mutated group, and TPX2 expression positively correlated with TP53 expression in HCC. Donehower et al. reported that a mutant TP53 RNA expression signature was correlated with impaired clinical outcomes in various tumors (Donehower et al. 2019), which further confirmed the prognostic power of TPX2 in HCC.

TIMER 2.0 analysis revealed strong positive correlations between TPX2 expression and the infiltration abundance of immune cells. A previous study reported that reduced TPX2 expression in HCC-infiltrating CD8 + T cells and downregulated TPX2 could restrict the anticancer effect of CD8 + T cells in HCC, whereas such antitumor activity could be enhanced by TPX2 overexpression (Wang et al. 2022a). TPX2-associated immune infiltration participates in the inhibitory effect of miR-29c on the malignant phenotype of HCC cells (Wang et al. 2023b). In colorectal cancer, TPX2 is closely associated with tumor-infiltrating lymphocytes (Guo et al. 2023). These findings suggest the involvement of TPX2 in the tumor immune microenvironment of HCC. In addition, hypoxia is a hallmark of solid tumors, especially HCC (Bao et al. 2021) and is one of the central modulators shaping the immune context of the tumor microenvironment (Suthen et al. 2022). For example, hypoxia can mediate the formation of an immunosuppressive state in the tumor microenvironment of HCC (Suthen et al. 2022). TPX2 was found to be positively correlated with the cellular response to hypoxia. This confirmed the association between TPX2 and immune infiltration. In addition to the cellular response to hypoxia, TPX2 positively correlated with multiple carcinogenesis-related pathways, such as the PI3K/AKT/mTOR pathway, tumor proliferation signature, and MYC targets. For example, the PI3K/AKT/mTOR pathway is a crucial intracellular signaling pathway linked to various aspects of cellular functions and is considered a unique pathway vital in regulating the cell cycle (Tewari et al. 2022). Targeting the PI3K/AKT/mTOR pathway has been proposed as an option for cancer prevention and intervention (Tewari et al. 2022). Such correlations indicate that these carcinogenesis-related pathways may be activated in HCC with TPX2 expression.

Additionally, we found that TPX2 exerts its biological functions in HCC in the form of functional modules, which could be explained by the close interactions and positive correlations with their co-expressed genes. The co-expressed genes were mainly implicated in cell cycle-related biological processes, such as mitotic nuclear division, chromosome segregation and cell division biological processes. This was consistent with results obtained from analyses for TPX2 only, that is, TPX2 showed strong positive correlations with pathways, including tumor proliferation signature, G2M checkpoints, DNA damage repair, and DNA replication. These results further confirmed that TPX2 was associated with tumor growth of HCC, and this might be achieved by interacting with genes related to cell proliferation. Based on TPX2 and its co-expressed genes, clinical prognostic models were established to predict the prognosis of patients with HCC, which performed better (AUC = 0.801, 0.725, and 0.711 for 1-, 3-, and 5-year survival, respectively) than the prognostic value of TPX2 alone (AUC = 0.73, 0.668, and 0.654 for 1-, 3-, and 5-year survival, respectively). These results further emphasize the functional modulatory activity of TPX2 in HCC.

**Fig. 11:**
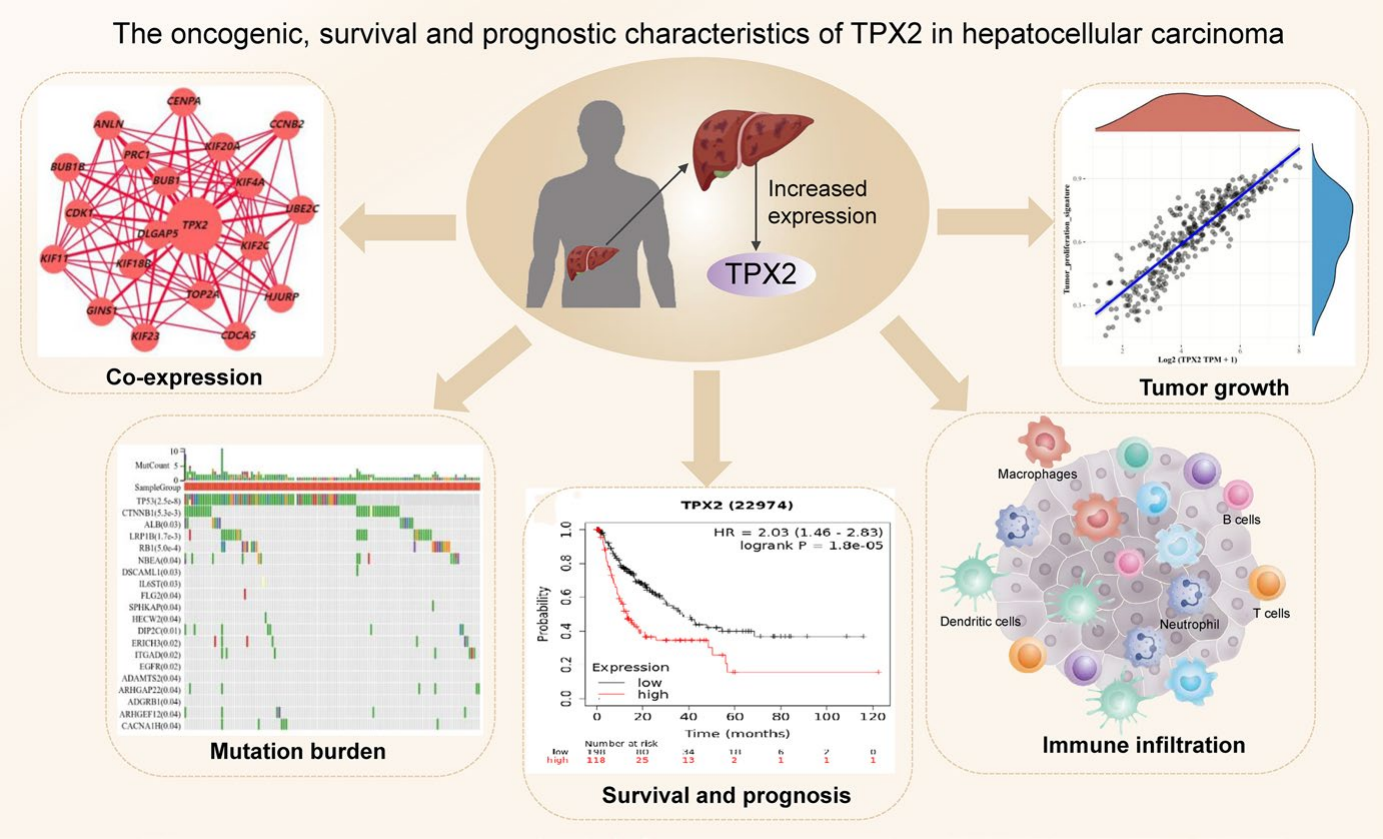
The conclusive figure (graphical abstract) for illustrating the main findings of this study.

The oncogenic, survival, and prognostic characteristics of TPX2 in HCC are summarized in Fig. 11. However, several limitations should be admitted. The exact biological functions of TPX2 and its co-expressed genes have not been explored in HCC using functional experiments. Moreover, whether the dysregulation of TPX2 was regulated by any epigenomic modifications or not, it is now unclear. In-depth elucidation of the regulatory mechanism for TPX2 expression and the exact biological functions of TPX2 in HCC are the focus of our future research.

## Conclusion

In conclusion, the results of this novel bioinformatic approach for analyzing the expression and prognostic significance of TPX2 in HCC suggest that it may be a potential target for the treatment of HCC. However, the results of this analysis need to be validated by corresponding basic and clinical studies to further clarify the molecular mechanism of the TPX2 gene in the survival prognosis of patients with HCC and its clinical significance.

## Abbreviations

TPX2: Targeting protein for Xenopus kinesin-like protein 2
HCC: Hepatocellular carcinoma
UALCAN: The University of ALabama at Birmingham CANcer data analysis Portal
GEPIA: Gene Expression Profiling Interactive Analysis
TIMER: Tumor Immune Estimation Resource
HCCDB: Integrative Molecular Database of Hepatocellular Carcinoma
HPA: Human Protein Atlas
ACLBI: Assistant for Clinical Bioinformatics
TCGA: The Cancer Genome Atla
GTEx: Genotype Tissue Expression
GEO: Gene Expression Omnibus
EGA: European Genome-Phenome Archive
GSEA: Gene set enrichment analysis
OS: Overall survival
PFS: Progression-free survival
DSS: Disease-specific survival
RFS: Recurrence-free survival
HR: Hazard ratios
CI: Confidence intervals
IHC: Immunohistochemistry
LASSO: Least absolute shrinkage and selection operator
ROC: Receiver operator characteristic
EMT: Epithelial-mesenchymal transition
